# Transient magnetic gratings on the nanometer scale

**DOI:** 10.1063/4.0000017

**Published:** 2020-09-08

**Authors:** D. Weder, C. von Korff Schmising, C. M. Günther, M. Schneider, D. Engel, P. Hessing, C. Strüber, M. Weigand, B. Vodungbo, E. Jal, X. Liu, A. Merhe, E. Pedersoli, F. Capotondi, J. Lüning, B. Pfau, S. Eisebitt

**Affiliations:** 1 Max-Born-Institute for Nonlinear Optics and Short Pulse Spectroscopy, 12489 Berlin, Germany; 2 Zentraleinrichtung Elektronenmikroskopie (ZELMI), Technische Universität Berlin, 10623 Berlin, Germany; 3 Helmholtz-Zentrum Berlin für Materialien und Energie, 12489 Berlin, Germany; 4 Sorbonne Université, CNRS, Laboratoire de Chimie Physique–Matière et Rayonnement, LCPMR, 75005 Paris, France; 5 Elettra-Sincrotrone Trieste, Basovizza, 34149 Trieste, Italy; 6 Institut für Optik und Atomare Physik, Technische Universität Berlin, 10623 Berlin, Germany

## Abstract

Laser-driven non-local electron dynamics in ultrathin magnetic samples on a sub-10 nm length scale is a key process in ultrafast magnetism. However, the experimental access has been challenging due to the nanoscopic and femtosecond nature of such transport processes. Here, we present a scattering-based experiment relying on a laser-induced electro- and magneto-optical grating in a Co/Pd ferromagnetic multilayer as a new technique to investigate non-local magnetization dynamics on nanometer length and femtosecond timescales. We induce a spatially modulated excitation pattern using tailored Al near-field masks with varying periodicities on a nanometer length scale and measure the first four diffraction orders in an x-ray scattering experiment with magnetic circular dichroism contrast at the free-electron laser facility FERMI, Trieste. The design of the periodic excitation mask leads to a strongly enhanced and characteristic transient scattering response allowing for sub-wavelength in-plane sensitivity for magnetic structures. In conjunction with scattering simulations, the experiment allows us to infer that a potential ultrafast lateral expansion of the initially excited regions of the magnetic film mediated by hot-electron transport and spin transport remains confined to below three nanometers.

## I. INTRODUCTION

The motivation to understand and control optically driven magnetization dynamics, in particular on the ∼10 nm length scale, is twofold. On the one hand, it represents the intrinsic spatial scale of fundamental properties of metallic magnetic systems governing ultrafast demagnetization and all-optical switching: for instance, with respect to interlayer coupling^1–3^ and diffusion lengths of laser-excited (spin-polarized) electron currents in layered magnetic systems,^4–7^ as well as materials exhibiting lateral chemical^8^ or magnetic^3,4^ heterogeneities. On the other hand, optical control of magnetic order on the ∼10 nm scale also has a strong technological relevance to achieve competitive bit sizes in potential future all-optical data storage applications. Some progress in nanoscale localization has been demonstrated in the field of heat-assisted magnetic recording in granular FePt layers by near-field laser enhancement.^9^ Plasmonic gold antennas were used for all-optical switching of nanoscale areas of CoFeTb alloys, however without resolving any time dependence and facing challenges due to chemical heterogeneities.^10^ Laterally inhomogeneous excitation caused by microstructuring of the sample was utilized in time-resolved photo-emission microscopy to reveal picosecond dynamics of all-optical switching^11^ and in a time-resolved Fourier transform holography experiment to image the femtosecond demagnetization of a magnetic domain pattern with a spatial resolution <70 nm.^12^ Plasmonic enhancement of the optical driving field via nanorods^13^ or gratings^14^ revealed a significant increase in the demagnetization amplitudes in all-optical Kerr studies. All-optical scattering techniques based on the Kerr effect have been employed to extract magnetic form factors and present a powerful and non-destructive tool to investigate magnetization of sub-micrometer-sized magnetic particles.^15^ However, to resolve lateral, non-local, and ultrafast spin dynamics in typical transition-metal-based magnetic samples exhibiting mean-free path lengths on the order of only several nanometers,^16,17^ a new experimental approach is needed providing the spatial sensitivity required.

Here, we present a femtosecond, resonant small-angle x-ray scattering (SAXS) experiment in the extreme ultraviolet spectral range (XUV) yielding spatiotemporal information of the evolving magnetization on a few nanometers lateral length and femtosecond timescales. An array of metallic nanogratings with a carefully chosen range of periodicities is patterned directly on top of a magnetic multilayer. Optical excitation transfers the shape of the metallic gratings into spatial patterns of time-dependent electro-optical (EO) and magneto-optical (MO) functions of the thin-film magnetic sample. Higher-order diffraction peaks from the evolving alternating magnetized and demagnetized areas are probed simultaneously in a single measurement via XUV magnetic circular dichroism (MCD). The interferometric approach allows for sub-wavelength sensitivity to lateral spatial changes of the magnetization in response to the optical excitation. We apply the approach to a ferromagnetic Co/Pd multilayer in the context of ultrafast optical demagnetization. We determine an upper boundary of ≈3 nm for a potential lateral increase in the initially optically excited area due to hot-electron transport.

The presentation of our results and analysis is organized into two parts: first, we introduce details of the investigated sample followed by a comprehensive analysis of the static scattering pattern, allowing us to determine exact dimensions and the relevant elemental composition of the metallic and magnetic domain grating. Second, we model the emergence of a transient nanoscale grating in real space after optical excitation and compare the corresponding transient diffraction intensities in reciprocal space with our experimental results. In particular, we demonstrate how qualitative differences in the response of the forbidden second-order diffraction intensities encode changes of the lateral magnetization on a nanometer scale.

## II. SAMPLE DESIGN

The investigated magnetic multilayer Al_2_O_3_(3)/Al(2)/Pd(3)/[Co(0.4)/Pd(0.2)]_30_/Al(3) was grown by magnetron sputtering on a ${\text{Si}}_{3}N_{4}$(30) membrane supported by a silicon frame (layer thickness in nm). The magnetic heterostructure exhibits a perpendicular magnetic anisotropy such that the magnetization direction is parallel or antiparallel to the surface normal. Prior to the experiment, the magnetic film was exposed to a decreasing oscillatory out-of-plane magnetic field to ensure a multi-domain state of ferromagnetic domains. Subsequently, an in-plane field was applied to align the domains into stripes with an orientation of 45° with respect to the membrane edges, leading to localization of the diffracted intensity in reciprocal space.

**FIG. 1.**
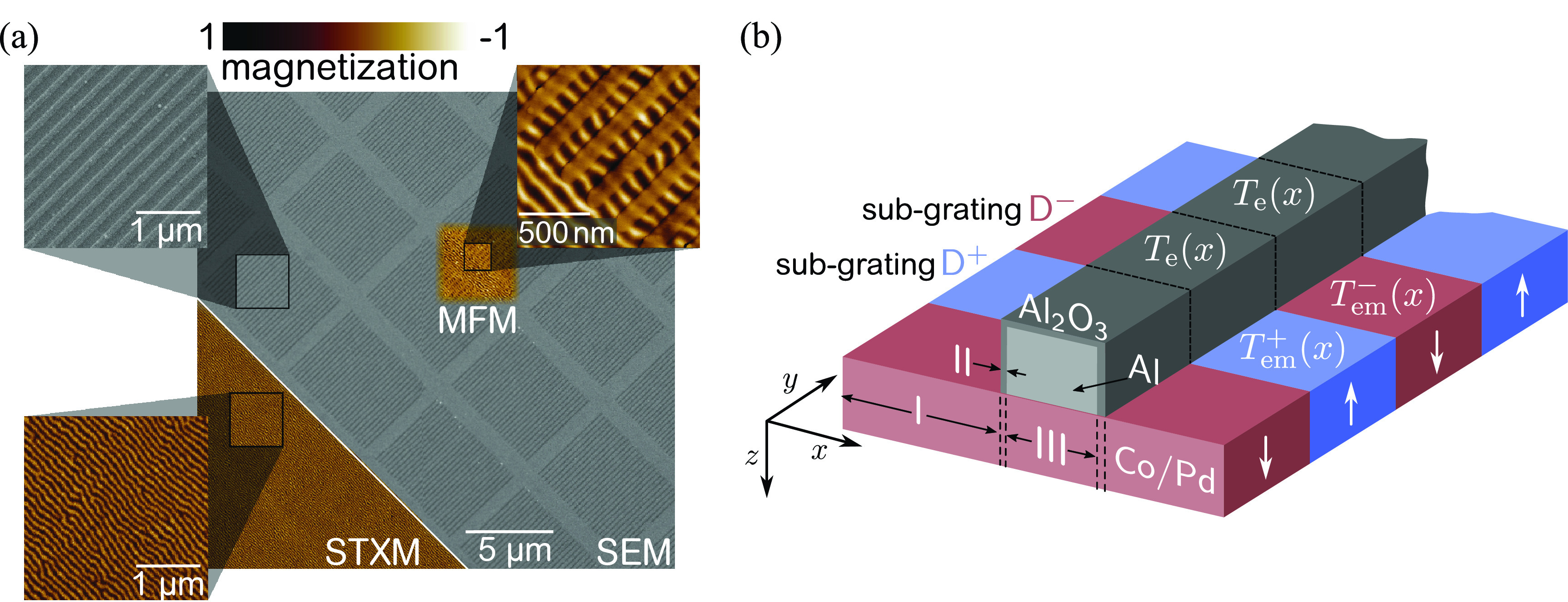
(a) Scanning electron microscope (SEM) image of a part of the nanostructured membrane displaying the ${\text{Si}}_{3}N_{4}$ membrane (light gray) and the Al grating bars (dark gray), arranged in several grating units. The magnified section in the upper left corner shows individual Al grating bars (light gray) with a width of about 100 nm. The remaining insets show the nanoscale domain pattern of the magnetic film measured via STXM at the Co L_3_ edge and by MFM. (b) Schematic of the composition of the two grating types, D^±^ defined by the grating functions $\left(T_{\text{em}}^{\pm }(x)\cdot T_{e}(x)\right)$ along antiparallel aligned magnetic domains, with $T_{e}(x)$ and $T_{\text{em}}^{\pm }(x)$ being the static electronic grating of the Al bar and the induced grating in the magnetic film, respectively. Both $T_{\text{em}}^{\pm }(x)$ and $T_{e}(x)$ are defined in three chemically and topographically different regions (I to III) along the *x*-direction.

Directly on top of the magnetic multilayer, we fabricated Al gratings via electron-beam lithography. Upon excitation with a pump laser, the Al gratings induce a patterned excitation profile with high contrast, due to the material's strong reflection and absorption of the optical pump while showing a high transmissivity of the XUV probe pulses. To separate the scattering signals from the magnetic domains and the metallic grating, the grating bars are oriented orthogonal to the magnetic stripe domains, as shown in Fig. 1(a). This geometry allows us to simultaneously measure the purely magnetic response of the multilayer by following the diffracted intensity of the magnetic domains.^18^ The entire grating structure covers a 50 *μ*m × 50 *μ*m membrane window and consists of 45 individual grating units with a size of 5 *μ*m × 6 *μ*m each, as seen in Fig. 1(a). The periodicity of the grating unit is systematically varied as follows (in nm): (A) 221, (B) 225, (C) 229, (D) 234, (E) 238, (F) 243, (G) 247, (H) 252, and (I) 256. For each of the nine periodicities, five identical units are part of the entire structure. Note that in the experiment, the entire grating structure is illuminated, and, hence, all grating units contribute to the diffraction signal at the same time. Each unit consists of 26 to 28 Al bars with an average linewidth and a height of 96(2) nm and 40 nm, respectively. We assume the thickness of the oxidation layer of the Al bars' surface and edges of ∼3 nm.^19^ By fixing the linewidth of the Al bars but continuously increasing the gratings' periodicities, we systematically vary their line-to-space ratio (LSR). As described in detail below, the effective LSR varies from slightly below to slightly above unity, causing a strong suppression of the second-order diffraction peaks. As a result, already small LSR changes of the transiently induced gratings will lead to a significant loss or increase in second-order intensities depending on the exact deviation of the Al grating's LSR from unity. We, thus, expect a qualitatively different transient response of the simultaneously probed gratings with varying LSR. The design of the scattering experiment to operate in the vicinity of a symmetry forbidden diffraction peak is the key to interferometrically achieve sub-wavelength spatial sensitivity, allowing us to detect subtle lateral spatiotemporal changes.

Post-inspection of the sample by magnetic force microscopy (MFM) and by scanning transmission x-ray microscopy (STXM) at the L_3_ edge of Co (carried out at the MAXYMUS beamline of the synchrotron-radiation facility BESSY II) reveals an undamaged metal grating and a fully functional magnetic layer [Fig. 1(a)] and allows us to rule out a free-electron laser (FEL)- or pump-induced modification of the sample.

## III. EXPERIMENT

**FIG. 2.**
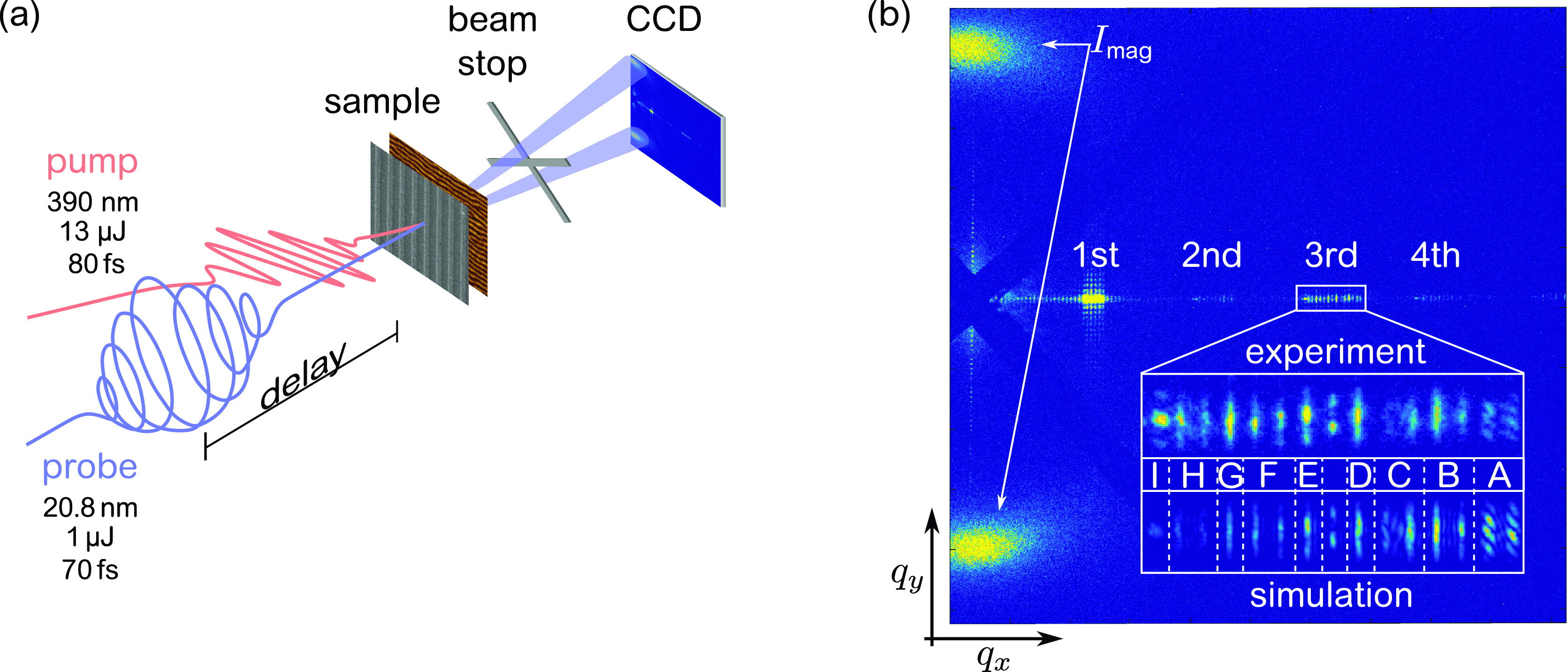
Sketch of the optical pump—XUV-probe scattering experiment with the corresponding diffraction pattern recorded at the FEL facility FERMI. (a) The sample is excited by optical p-polarized laser radiation. The induced dynamics are probed by time-delayed circularly polarized femtosecond XUV pulses under normal incidence. which are tuned to the resonance of the Co M edges. (b) Typical diffraction pattern from a non-excited sample. The CCD image captures scattering intensity up to the fourth diffraction order along *q_x_*, originating from the vertically aligned static Al gratings, and the first-order magnetic domain diffraction along *q_y_*, originating from the horizontally aligned magnetic domains.

The experiment was carried out at the FEL facility FERMI, in Trieste, Italy, using the DiProI endstation in an optical-pump—XUV-SAXS-probe scheme in transmission geometry [Fig. 2(a)]. The spatially patterned excitation was induced by 80 fs full width at half maximum (FWHM) optical laser pulses centered at a wavelength of 390 nm. The optical pulse was collimated to a size of 260 *μ*m × 270 *μ*m (FWHM), and its pulse energy was limited to 13 *μ*J. Its linear polarization direction was set perpendicular with respect to the orientation of the Al bars to increase the deposited energy within the uncovered magnetic film.^14^ Time-delayed, circularly polarized, 70 fs FWHM XUV pulses were tuned to the Co M edge resonances at a wavelength of 20.8 nm (59.6 eV) to probe the time evolution of the laser-induced transient magnetic gratings exploiting the XUV MCD contrast.^20^ A FEL pulse energy of 1 *μ*J with a footprint of 240 *μ*m × 230 *μ*m (FWHM) corresponding to a fluence of $2.2\;\text{mJ}/cm^{2}$ was chosen to avoid FEL-induced non-reversible changes or rearrangement of the magnetic domain pattern.^21,22^ A cross-shaped beamstop in front of the detector blocks the intense direct beam and the strong scattering from the edges of the membrane window. The detector, placed 90 mm behind the sample, is a 27.6 mm × 27.6 mm (2048 pixel × 2048 pixel) large in-vacuum, back-illuminated charge-coupled device (CCD). An off-axis geometry of the detector by 10° allows us to detect the grating diffraction up to the fourth order along *q_x_*.

Figure 2(b) shows the static diffraction pattern integrated over 500 XUV pulses at a repetition rate of 10 Hz. The pattern contains the first-order scattering from the aligned magnetic stripe domains along *q_y_* and the static diffraction from the Al gratings along *q_x_*. As all gratings with different periods are illuminated simultaneously, we observe a group of diffraction peaks for each order [Fig. 2(b)].

We simulate the scattering intensities based on the far-field Fraunhofer approximation using the binary grating design as real-space input. This very simplified approach allows us to clearly assign each diffraction peak to a particular grating periodicity [inset in Fig. 2(b)]. The simulation even reproduces the internal intensity difference between different grating units caused by the designed sequence of units and finite dimension of the sub-grating areas. Diffraction peaks that are partly composed of a superposition from gratings with different periodicities are not used in the analysis [e.g., scattering intensity positioned in *q*-space between the third order diffraction of grating E and D in the inset of Fig. 2(b)]. However, we find a mismatch with respect to the absolute diffraction intensities, which we discuss in the following.

## IV. RESULTS

### A. Quantitative simulation of the static diffraction

The index of refraction of a magnetized material probed by resonantly tuned, circularly polarized XUV, or soft-x-ray radiation is typically expressed as^20,23^

$$n^{\pm }(\lambda )=1-[\delta (\lambda )\pm \Delta \delta (\lambda )]+i[\beta (\lambda )\pm \Delta \beta (\lambda )].$$ (1)

Here, δ(λ) and β(λ) denote the electro-optical (EO) constants describing dispersion and absorption of the material for unpolarized radiation with wavelength *λ*. The magneto-optical (MO) constants Δβ and Δδ correct the EO constants if circular polarization is used. The alternating sign refers to either parallel or antiparallel alignment of the magnetization with respect to the wave vector of the incident radiation.

Our refined model is based on a sample structure as schematically shown in Fig. 1(b) comprising the Al_2_O_3_/Al grating with a grating vector along the *x*-axis and the alternating magnetic domains along the *y*-axis. As the sample is otherwise uniform with respect to sample topography and the chemical composition along the *y*-axis, the resulting scattering contrast along *q_y_* is only given by the dichroic MO constants. On the other hand, the Al_2_O_3_/Al gratings give rise to diffraction along *q_x_*, based on the static EO contrast along *x*. In the following, we will focus our analysis on the sample structure along the *x*-direction and reduce our model to a quasi-one-dimensional representation. Therefore, we divide the sample model into two sub-gratings D^±^ with XUV transmission functions $T^{\pm }$ referring to “up” (+) or “down” (−) magnetization of the multilayer underneath the Al_2_O_3_/Al grating. Again, based on the Fraunhofer approximation, the Fourier components of these transmission functions are directly related to the scattering intensities. For a layered sample, the transmission function is given by

$$T^{\pm }(x)\propto \;\text{exp}\left(\frac{2\pi i}{\lambda }\sum_{k}d_{k}(x)n_{k}^{\pm }(\lambda )\right),$$ (2)

where $d_{k}(x)$ denotes the thickness of the layer of material *k* and $n_{k}^{\pm }(\lambda )$ its (potentially dichroic) refractive index.

**FIG. 3.**
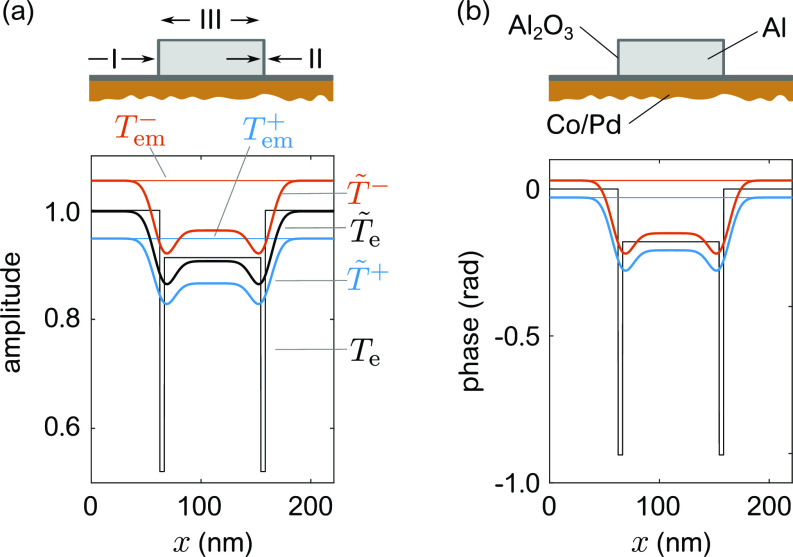
Cross section along the *x*-direction of the sample as defined in Fig. 1(b) and corresponding static amplitude (a) and phase (b) gratings at a wavelength of 20.8 nm (59.6 eV). At the Co resonance, the transmission function is given by the electronic, $T_{e}(x)$, contribution of the Al bar and the electronic/magnetic, $T_{\text{em}}^{\pm }(x)$, contributions of the complex index of refraction along the D^+^ and D^−^ sub-grating. We differentiate three distinct sections with different chemical compositions and topography: (I): the uncovered magnetic film denoted as space, (II) the Al_2_O_3_ line edge, and (III) the area covered by Al denoted as line.

We calculate the diffraction intensity $I(q_{x})$ by coherently adding the Fourier coefficients of the two sub-gratings,

$$I(q_{x})=|\left(f{\tilde{T}}^{+}\left(x\right)\right)\left(q_{x}\right)+\left(f{\tilde{T}}^{-}\left(x\right)\right)\left(q_{x}\right){|}^{2},$$ (3)

where $\tilde{T}$ denotes the transmission functions corrected for the finite wavelength of the XUV radiation (cf. Appendix A). The result for *T*^+^ (blue) and *T*^−^ (red) of our sample model along *x* is plotted in Fig. 3, separately for their amplitude (a) and phase (b). In order to separate contributions from the grating mask and the functional magnetic layer, we decompose the transmission functions into $T^{\pm }(x)=T_{\text{em}}^{\pm }(x)\cdot T_{e}(x)$, with $T_{\text{em}}^{\pm }(x)$ referring to the magnetic film with EO and MO contributions and $T_{e}$ referring to the Al_2_O_3_/Al grating providing exclusively EO contrast. In the static case, $T_{\text{em}}^{\pm }(x)$ (thin lines) are homogeneous along *x*. In contrast, the pure electronic grating $T_{e}(x)$ (black lines) consists of three distinct sections with different chemical compositions and topography: (I) the uncovered magnetic Co/Pd film (between the Al bars denoted as “space”), (II) the oxidized Al line edges, and (III) the Al covered magnetic film denoted as “line.” As shown in Figs. 3(a) and 3(b), the transmission function of section (II) shows significant lateral modulation of amplitude and phase due to the presence of Al_2_O_3_ at the line edges.

**FIG. 4.**
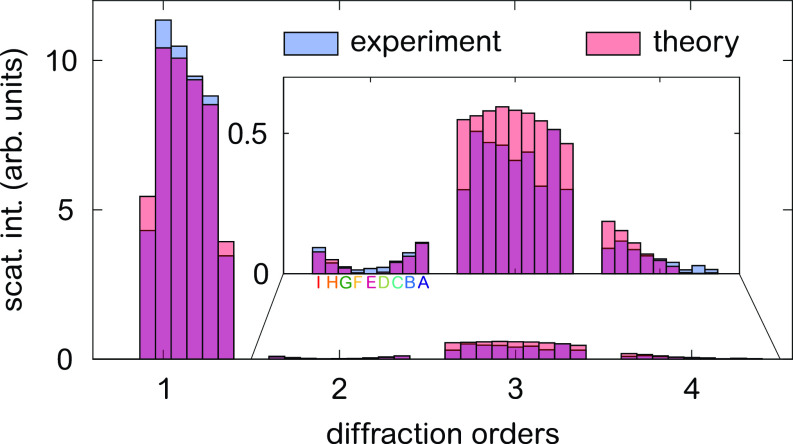
Comparison between experimentally observed (blue bars) and calculated (red bars) static Fourier coefficients based on Eq. (3) for the first four diffraction orders and all nine grating periodicities at a wavelength of 20.8 nm (59.6 eV).

To compare the calculated $I(q_{x})$ and measured diffraction intensities, we evaluate Eq. (3) for the first four diffraction orders and show the diffraction intensities in Fig. 4. (For more details regarding the calculation and for a list of the used EO and MO constants, we refer the reader to Appendix A.) The histogram shows a good quantitative agreement between the measured (blue) and calculated (red) Fourier coefficients. The significant suppression of the even orders is well reproduced and indicates that the variation of the Al grating periodicities indeed is centered around a LSR value of unity. In order to further corroborate our real-space model of our sample, we performed an additional measurement at an off-resonant wavelength of λ=17.8 nm (69.5 eV). The analogous analysis again yields a good agreement (cf. Appendix B).

In summary, we are able to quantitatively describe the simultaneous diffraction from gratings with different periodicities in the first four diffraction orders based on the structure of the sample, literature values for the complex refractive index, and its dichroic contribution. We will now use this model to analyze the transient diffraction data and infer the optically induced lateral magnetization changes.

### B. Transient diffraction after optical excitation

**FIG. 5.**
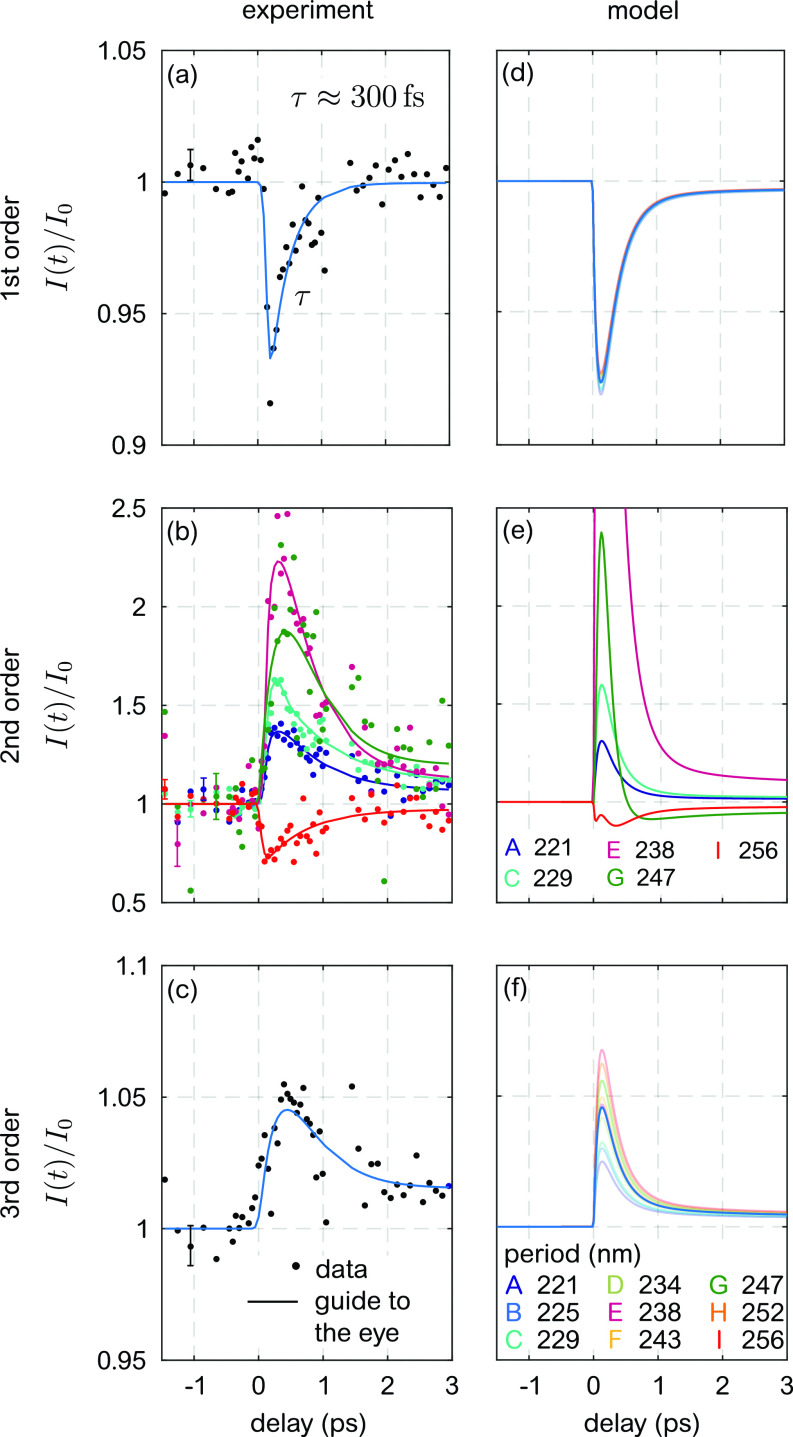
Comparison between recorded, (a)–(c), and simulated, (d)–(f), diffraction intensity for the first three diffraction orders. For the reason of clarity and comprehensibility, we only plot the response of the second order for selected gratings A, C, E, G, and I. Because of a weak dependence between grating periodicity and scattering intensity observed in the first and third order, we only show the corresponding measured data averaged over all periodicities. The solid lines in (a) to (c) serve as guides to the eye. The error bars shown in (a) for the first and in (c) for the third order correspond to the standard error, while in (b), the error bars of the response for the individual gratings are calculated as the standard deviation of the data points before time delay zero.

The experimental results of the optical pump—XUV-probe experiment are shown in Fig. 5, panels (a)–(c). We plot the normalized laser-driven evolution of the diffraction intensity, $I(t)/I_{0}$, for the first three diffraction orders along *q_x_* as a function of time delay between −1 ps and 3 ps. *I*_0_ is the average diffracted intensity for *t *<* *0. All three diffraction orders show a distinct and individual transient behavior. For the first and third order, we have averaged the response from gratings with different periodicities as they do not show any pronounced periodicity dependence. While we observe a decrease in the scattered intensities after optical excitation in the first order, we observe an increase in the third-order intensities. Most interestingly, the second-order diffraction intensities display a very complex ultrafast evolution with qualitative differences for gratings with different periodicities. This very distinct qualitative response of the forbidden second-order intensity for periodicities only differing by 4–5 nm suggests a pronounced sensitivity to transient changes of the LSR ratio. To understand the details of the ultrafast response and in order to extract quantitative information on the nanometer-scale lateral evolution of laser-manipulated magnetization, we apply the diffraction model developed and validated in the static case in Sec. IV A to the dynamic case in the following.

#### 1. Simulation of the excitation pattern

To model the ultrafast dynamics as reflected in the diffraction intensities, we first require knowledge about the spatially structured excitation. To quantify the excitation profile, we performed a 2D simulation of the electric-field distribution using the RF module of the commercial-grade simulator COMSOL Multiphysics based on the finite-element method. Along the *x*-axis of the sample, we observe a periodic modulation of the optical excitation with almost unexcited areas below the Al bars [Fig. 1(b), area III], full excitation in the uncovered areas (I), and a local field enhancement at the sharp edges of the Al bars (II) (see Appendix C for details). This modulation gives rise to an additional transient scattering contrast acting as a fingerprint of the spatially inhomogeneous response of the sample. As the periodicity of the static Al grating is identical to the induced transient grating in the magnetic multilayer, their diffraction peaks share the same scattering vectors. In Sec. IV B 2, we show how the static and transient contributions to the scattering amplitude are combined in the model.

#### 2. Transient magneto- and electro-optical response

The optical excitation of the sample leads to a perturbation of the electronic system and, as a consequence, to ultrafast demagnetization. To verify that potential dynamic changes of EO constants of the Al/Al_2_O_3_ mask do not influence our measured signals, we performed additional pump–probe measurements at the XUV wavelength of λ=17.8 nm (69.5 eV photon energy), i.e., away from any resonance in our sample. In these measurements, we do not detect any appreciable transient response in the diffraction spots, corroborating the starting hypothesis that laser-induced changes of the EO constants in the Al_2_O_3_/Al mask are negligible (see Appendix B for further details). We can, therefore, focus on analysis of the sample's transient response to the magnetized Co layers within the Co/Pd multilayers and neglect electronic changes in all other materials.

**FIG. 6.**
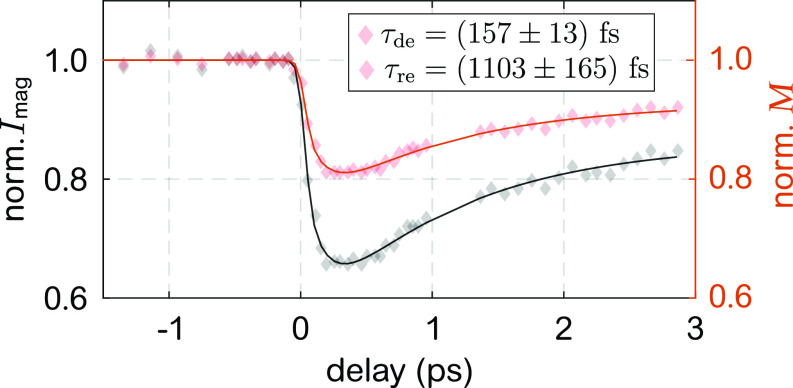
Transient laser-induced first-order magnetic domain scattering $I_{\text{mag}}$ and extracted magnetic response *M*. The temporal evolution of *M* and its maximum loss during ultrafast demagnetization of 19% are utilized to model the transient MO constants Δδ(t) and Δβ(t). The standard deviation of data points before time delay zero is <1%.

The intrinsic timescales and amplitudes of the laser-driven MO dynamics can be directly extracted from the magnetic-domain scattering intensity [recorded in the vertical direction of the diffraction pattern, see Fig. 2(b)], which is proportional to the domain's magnetization *M*(*t*) squared.^18^ The scattering intensity after excitation (black symbols) and the corresponding normalized magnetization $M(t)/M_{0}$ (red symbols) are displayed in Fig. 6. A double-exponential fit yields a maximum demagnetization of 19 % and de- and remagnetization time constants of (157 ± 13) fs and (1.1 ± 0.2) ps, respectively (compare Appendix D for more details). Later, we use $M(t)/M_{0}$ in our model describing the transient modulation of the MO constants of Co.

As we show next, we can directly use the transient intensity changes of the first-order diffraction ($I_{1}(t)/I_{0}$) to infer the dynamics of the EO constants of the Co/Pd film. In this diffraction order, the induced changes of the diffraction amplitudes from the transient magnetic sub-gratings D^+^ and D^−^ lead to diffraction intensity changes that almost cancel each other due to an inverted grating contrast in the oppositely magnetized domains. (A small asymmetry in the individual diffraction intensity amounts to changes <0.5 %.) In both domain types, the MO constants change by the same magnitude, but with opposite signs. As expected for the response of the electronic system, the signal drops instantaneously with respect to our time resolution of approximately 110 fs and relaxes with a time constant of approximately 300 fs [cf. Fig. 5(a)].^24^ In particular, this response is very different from the magnetization dynamics shown in Fig. 6, further corroborating our assumption that the first-order diffraction peaks provide a direct measure of the ultrafast evolution of the electronic perturbation.

Using the temporal dependence of the EO and MO functions extracted from the data in Figs. 5(a) and 6, respectively, we incorporate the spatiotemporal magnetic and electronic responses of Co directly into time-dependent scaling functions $\alpha_{\text{EO}}(x,t)$ and $\alpha_{\text{MO}}(x,t)$ of the EO and MO constants of Co, respectively. That is, we replace the optical constants of Co by: $\Delta \beta_{\text{Co}}(x,t)=\alpha_{\text{MO}}(x,t)\Delta \beta_{\text{Co}}$ and $\Delta \delta_{\text{Co}}(x,t)=\alpha_{\text{MO}}(x,t)\Delta \delta_{\text{Co}}$ as well as $\beta_{\text{Co}}(x,t)=\alpha_{\text{EO}}(x,t)\beta_{\text{Co}}$ and $\delta_{\text{Co}}(x,t)=\alpha_{\text{EO}}(x,t)\delta_{\text{Co}}$ [cf. Eq. (A1) in Appendix A]. In addition, we slightly simplify the excitation profile as follows: in the areas (III) covered by Al (“lines”), we assume no excitation, i.e., $\alpha_{\text{MO}}(\text{III},t)=\alpha_{\text{EO}}(\text{III},t)=1$. In the exited area (I) (“spaces”), we model $\alpha_{\text{MO}}(I,t)=M(t)/M_{0}$ and $\alpha_{\text{EO}}(I,t)=2-\sqrt{I_{1}/I_{0}}$ corresponding to a maximum change of 3.5% in agreement with the literature.^25^ The fivefold electric-field enhancement in sections (II) below the oxide-metal interface is modeled by a non-linear increase in the scaling factors: $\alpha_{\text{MO}}(\text{II},t)=4(\alpha_{\text{MO}}(I,t)-1)+1$ and $\alpha_{\text{EO}}(\text{II},t)=3.4(\alpha_{\text{EO}}(I,t)-1)+1$. The prefactors, which empirically model the non-linear response to the field enhancement, were found by fitting the third-order response in the model to the experimental findings.

#### 3. Dynamic transmission profiles

**FIG. 7.**
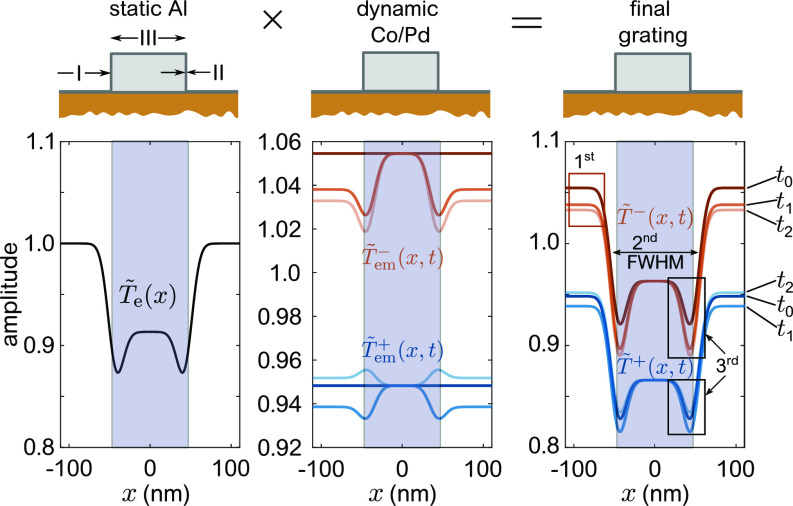
Temporal evolution of the amplitude part of the transmission function shown for a cross section of the sample. Left frame: static Al_2_O_3_/Al amplitude grating. Center frame: laser-induced EO and MO amplitude for three consecutive time delays, $t_{0}=-500\;\text{fs},\;t_{1}=60\;\text{fs},$ and $t_{2}=500\;\text{fs}$. Right frame: in the resulting amplitude grating, three distinct areas are identified (marked with boxes) where ultrafast changes take place. These changes lead to a predominantly decoupled modulation of the first, second, and third diffraction orders.

Figure 7 illustrates how we composed the transient transmission function of the sample in our model. We only show the amplitudes as the phases act analogously. As detailed above, the non-resonantly probed Al_2_O_3_/Al grating contrast remains static upon optical excitation. The central panel depicts the evolution of the EO and MO gratings induced in the Co/Pd multilayer for three time steps before and after excitation, at $t_{0}=-500\;\text{fs},\;t_{1}=60\;\text{fs}$, and $t_{2}=500\;\text{fs}$.

Before optical excitation, at *t*_0_, the Co MCD leads to a constant negative, +Δβ, or positive, −Δβ offset of ${\tilde{T}}_{\text{em}}^{\pm }(x,t)$ in the oppositely magnetized domains. Upon optical excitation, both types of domains respond differently. For down domains (${\tilde{T}}_{\text{em}}^{-}$), corresponding to $\alpha_{\text{EO}}(x,t)\beta_{\text{Co}}-\alpha_{\text{MO}}(x,t)\Delta \beta_{\text{Co}}$, a significant reduction of the amplitude is introduced, both by a laser-induced increase in $\alpha_{\text{EO}}(x,t)$ and decrease in $\alpha_{\text{MO}}(x,t)$. The situation changes for the up domains (${\tilde{T}}_{\text{em}}^{+}$), corresponding to $\alpha_{\text{EO}}(x,t)\beta_{\text{Co}}+\alpha_{\text{MO}}(x,t)\Delta \beta_{\text{Co}}$, where both effects act in the opposite way, i.e., the decrease in $\alpha_{\text{MO}}(x,t)$ compensates the increase in $\alpha_{\text{EO}}(x,t)$. As a result, we observe a transient reduction of the amplitude at short times (*t*_1_), when the EO effects dominate, followed by an increase above the initial amplitude value for later times (*t*_2_) with mainly MO contributions. The coherent sum of both sub-gratings is then dominated by the amplitude of the D^−^ sub-grating because it is subject to a larger contrast change.

The product of the static and dynamic contributions is shown in the right panel of Fig. 7 where we identify three characteristic features of the transmission function, which will directly and almost independently influence the scattering amplitude of the three different diffraction orders. First, the contrast between the excited and non-excited magnetic films is reduced, which mainly leads to a decrease in the first-order diffraction (marked with a red box in Fig. 7). Second, an expansion or contraction of the transverse width of the non-excited area (III) in the magnetic film will manifest itself as a transient change in the second-order signal because it is dominated by changes of the LSR. Third, the areas affected by electric field enhancement are subject to the largest changes of ${\tilde{T}}^{\pm }(x)$ (marked by black boxes in Fig. 7). The third order at large scattering vectors sampling small real-space frequencies is dominated by the localized excitation at the edges of the Al bars.

#### 4. Simulation of diffraction intensity

Panels (d)–(f) in Fig. 5 show the simulated transient intensity for the first three diffraction orders for all nine grating periodicities according to Eq. (3). Note that there is no further fit parameter and that all diffraction orders observed for gratings with different periodicities are calculated based on the same time dependence of the EO and MO constants. Also, note that the absolute y-axis scaling is the same for the data and the simulation. The intensity of the first diffraction order is dominated by the D^−^ sub-grating and decreases as the overall grating contrast between excited and non-excited regions drops after laser excitation. The weak dependence of the first order on the grating periodicity again justifies to average the measured response from the individual gratings [bold blue line in panel (d) of Fig. 5].

The third order mainly samples the response in the electric field-enhanced areas directly below the Al line edges (Fig. 7, black boxes). If we do not consider this edge effect in our model, the characteristic transient increase in the third diffraction order in the experiment cannot be reproduced. Again, the response is dominated by the D^−^ sub-grating, where the peak gradient increases after optical excitation. Similar to the first order, the grating periodicity dependence of the dynamics is only on the order of a few percent and we average the response over all gratings [bold blue line in panel (f) of Fig. 5].

Very similar to the experimental findings, the second diffraction order shows the most complex behavior: the time-dependent interplay between the static electronic contrast grating of the Al structure, ${\tilde{T}}_{e}$, and the transient electronic/magnetic grating in the Co/Pd multilayer, ${\tilde{T}}_{\text{em}}^{\pm }$, modulates the width of non-excited region III and, hence, effectively changes the LSR. For the D^−^ sub-grating, which again dominates the overall transient response, the simulation predicts an increase in the FWHM by only 1.3 nm independent of the grating periodicity. These very subtle changes, nonetheless, lead to the observed qualitatively different dynamic response for different grating periodicities. Gratings with an initial LSR greater than unity gain second-order intensity by the additional increase in the LSR. In contrast, gratings with an initial LSR smaller than unity approach a configuration with a forbidden second-order peak and, consequently, lose scattering intensity. We find good agreement between experimental data and the simulation; the changes are of a comparable magnitude, and the qualitative trend for the different gratings is well reproduced. With increasing periodicity, $I(t)/I_{0}$ increases and reaches a maximum for gratings E and F. Because our model assumes a perfect grating structure the changes diverge, while we measure finite changes for these two gratings in the experiment. For grating H, the changes approach zero and grating I shows a decreasing scattering amplitude after optical excitation. Finally, we would like to mention that we have performed the same experiment on a different Al grating array exhibiting identical periodicities but with slightly smaller Al bar line widths, i.e., with a different LSR. Here, we observed an increase in the second scattering order intensity for gratings A–C and a decrease for gratings E–I, again in agreement with our model (for more details, we refer to Ref. 26).

## V. DISCUSSION

It is important to note that our modeling does not include any energy transport by, e.g., hot electrons nor non-local magnetization processes such as superdiffusive spin transfer.^4^ The nanometer-scale confinement of the optical pump pulse as well as the large excitation gradients due to the field enhancement at the edge of the Al bars, however, may be considered as a likely cause for such lateral transport processes. Laser-driven energy transfer would lead to a lateral excitation pattern distinct from the Al grating: areas below the Al bars (lines) would become excited as well, effectively changing the dynamic LSR. Scattering intensities would change accordingly, as both, the electro- and magneto-optical functions would change due to transfer of excited electrons and their interaction with the spin system, leading to ultrafast demagnetization.^7,27^ Hence, the analysis of our experimental findings, in particular, the response of the second order, allows us to determine an upper limit for the effective range of potential lateral transport processes after the initial optical excitation.

As outlined above, already lateral changes on the order of 1 nm are sufficient to yield a qualitatively distinct second-order response for gratings with different periodicities. Hence, to determine an absolute value of potential ultrafast electron transport, the uncertainty of our experiment is determined by how well the initial excitation pattern is known, or in other words, how well the Al_2_O_3_/Al structure and its interaction with the optical excitation can be established. The great advantage of our technique based on measurements around a forbidden diffraction peak is that only the qualitative differences in the shape of the transient response already results in a very high spatial sensitivity. A statistical test evaluating data within the time range between 0.1 ps, and 1.0 ps, determines a confidence interval of >0.9973 or 3.0 *σ* for all nine gratings with which we can differentiate whether the diffraction efficiency of the second order increases or decreases after optical excitation (cf. Appendix E). Then, the corresponding spatial sensitivity is simply given by the absolute difference of successive periodicities of the Al mask around the effective LSR of 1, in our case 4–5 nm. Because our grating is symmetric, any electron transport would emanate from both interfaces, effectively doubling our sensitivity to ≲3 nm. Finally, we note that, because of the finite penetration depth of the optical excitation, transport processes may be more pronounced close to the surface. However, because we measure in a transmission geometry that would merely result in a quantitative but not qualitative change of the second-order response. Therefore, our experimental results together with our simulation suggest that non-local lateral processes transiently altering the local EO and MO constants during the optical demagnetization of a Co/Pd multilayer must be confined to less than 3 nm.

The spin-dependent electron inelastic mean free paths for transition metals have been calculated in the relevant energy range of up to 3 eV above the Fermi energy and vary approximately between 2 and 8 nm in Fe and Ni for majority carriers.^16^ This needs to be compared with the corresponding mean free path of minority electrons of only 1–2 nm.^16^ The resulting ratio of the transport characteristics between majority and minority carriers may be overestimated, though, as experiments on spin-dependent lifetimes have been reporting fairly moderate values in the range of 1–2 nm.^28,29^ Nonetheless, these numbers are in good agreement with a recent systematic study of spin currents in Co determining a mean free path of 3 nm in Co via magnetization-induced second-harmonic generation.^17^ In an earlier work based on magnetic small-angle x-ray scattering, we have reported larger maximum values for spin diffusion lengths.^30^ Here, ultrafast changes of the scattering angle were explained by an effective broadening of the magnetic domain interface of up to 20 nm (FWHM). It is important though, that this was shown to be a highly non-linear process and that the maximal value was determined for significantly stronger demagnetization amplitudes of approximately 70%, while for demagnetization amplitudes of 20%, as observed in the present study, no shift of the scattering angle could be detected. A second difference between the two experiments is the excitation wavelengths, namely, 800 nm photons vs 400 nm photons in the present work. This is worth mentioning as a very recent experimental work reported on more efficient demagnetization for longer excitation wavelengths and explained this by a wavelength dependence of both, the laser-induced heating of the electrons ($T_{e}\propto \lambda^{2}$) and the spatial distribution of the electromagnetic energy deposited into the multilayer sample.^31^ These considerations call for further systematic studies on non-local magnetization dynamics controlling the excitation density and wavelength in sample systems with an identical chemical composition as well as identical geometries and structures. We are confident that the method presented here is ideally suited for this task.

## VI. CONCLUSION

In conclusion, we have introduced a new method to determine ultrafast demagnetization dynamics on a nanometer length scale based on measurements of scattering intensities around a forbidden diffraction order in tailored grating geometries. We demonstrated that optically excited metallic nanometer-sized gratings on top of a ferromagnetic film induce nanoscale transient magnetic gratings on a femtosecond timescale. The temporal evolution of the measured first diffraction order stemming from the magnetic domains and the grating induced by the Al mask provides quantitative information about the time constant and amplitude of magnetization and the electro-optical dynamics of the multilayer structure, respectively. While the third order encodes information about the local enhancement at the Al grating edge, the suppressed transient second-order response determines the lines-to-spaces ratio with a nanometer accuracy. The latter allows us to determine an upper value for the effects of transport-mediated processes, which laterally smear out demagnetization patterns induced by a nanoscale localization of the excitation. For our experimental conditions with moderate excitation densities, we can exclude effective lateral transport processes within a range larger than 3 nm, in agreement with the literature. We note that our method to increase lateral sensitivity by using an artificially generated forbidden grating diffraction allows us to investigate lateral transport even in the absence of interfaces and beyond magnetic samples. We envision that similar experiments with tailored excitation profiles carried out in the soft x-ray spectral range will further push the limits of the spatial resolution benefiting from much smaller charge scattering of the near-field mask, allowing, e.g., for the study of spin-dependent electron transport in uniformly magnetized samples and through magnetic domain walls.

## Figures and Tables

**FIG. 8.**
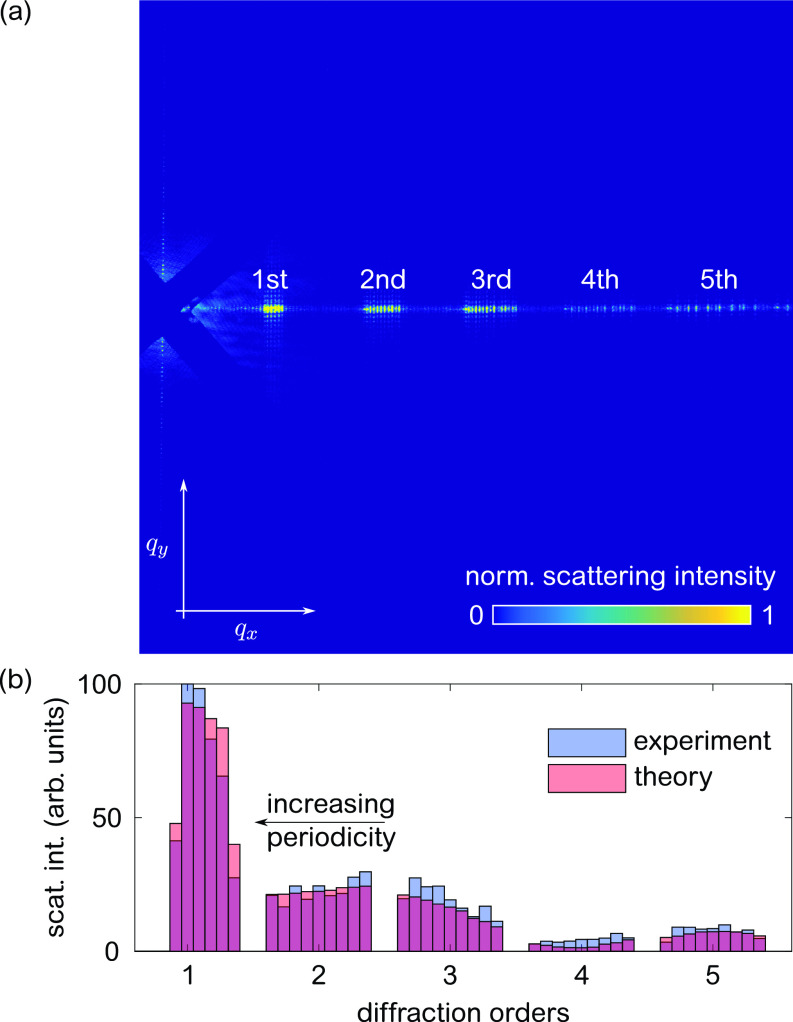
(a) Typical diffraction image of a non-excited grating sample recorded off-resonantly at a wavelength of ≈17.8 nm(69.5 eV). Due to the higher photon energy compared to the on-resonant measurement, scattering vectors up to the fifth diffraction order can be recorded. (b) Comparison between experimentally observed (blue bars) and calculated (red bars) off-resonant static Fourier coefficients for the first five diffraction orders and all nine gratings with varying periodicities. The striking difference between the off- and on-resonant observed Fourier coefficients (see, therefore, Fig. 4) is also well reproduced by our model.

**FIG. 9.**
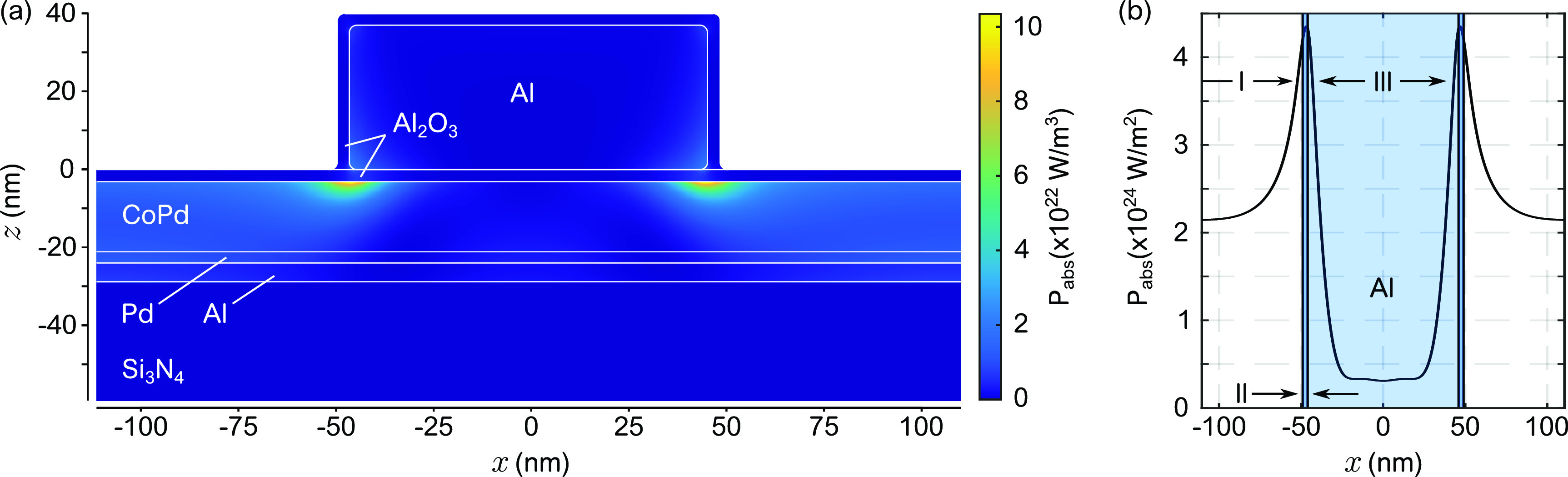
(a) Finite-element simulation of the 2D distribution of the absorbed power $P_{\text{abs}}$ within the cross section of grating A with a grating periodicity of 221 nm. The calculation is performed for a UV pump beam under normal incidence centered at λ=400 nm. The linear polarization is aligned perpendicular with respect to the Al lines and parallel to the *xz*-plane. The incident fluence is $6.5\;\text{mJ}/cm^{2}$. The magnetic film is modeled in an effective medium approach as a homogeneous CoPd alloy (cf. Appendix C). (b) Integration of $P_{\text{abs}}$ along the sample depth of (a). The bluish area marks the area covered by Al and the 3 nm wide Al_2_O_3_ layers.

**FIG. 10.**
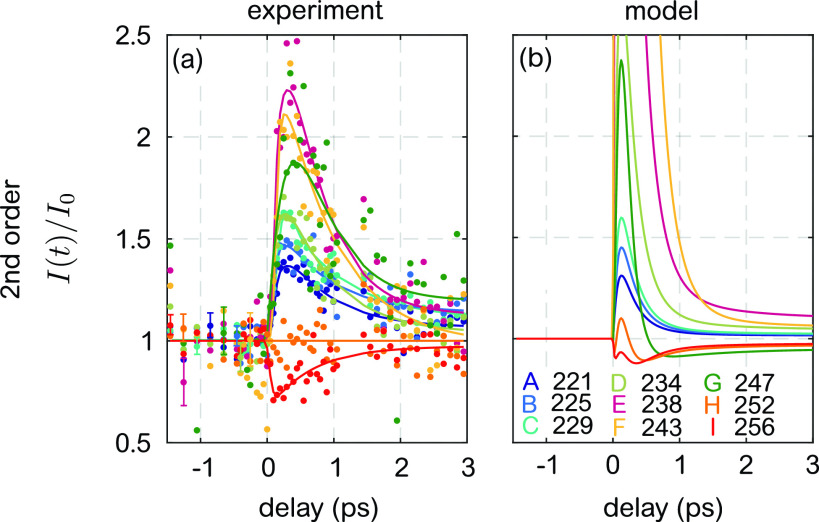
Measured (a) and modeled (b) transient diffraction intensity, $I(t)/I_{0}$, of the second order as a function of time delay for all gratings A–I.

**FIG. 11.**
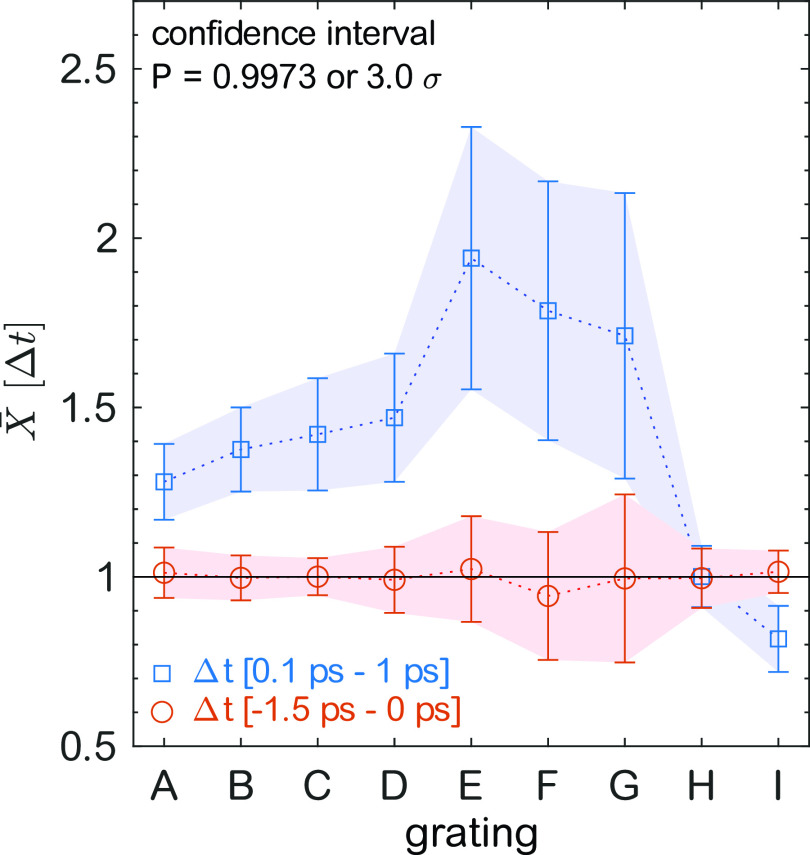
Statistical analysis of the transient diffraction intensity of the second order. We plot the mean value $\bar{X}[\Delta t]$ of $I(t)/I_{0}$ for time delays before optical excitation between −1.5 ps and 0 ps (red circles) and for time delays after optical excitation between 0.1 ps and 1.0 ps (blue squares). The error bars correspond to 3σ (0.9973) and determine the confidence interval with which we can claim that the second-order diffraction increases for gratings A–G and decreases for grating I after optical excitation.

## Data Availability

The data that support the findings of this study are available from the corresponding author upon reasonable request.

## APPENDIX A: QUANTITATIVE SIMULATION OF THE STATIC DIFFRACTION

In the following, we give more details on the calculation of the static diffraction pattern.

Using the index of refraction from Eq. (1) and neglecting the vacuum term $\text{exp}\;(-2\pi id\lambda^{-1})$, we obtain

$$\begin{array}{c} T^{\pm }(x)=\;\text{exp}\left(\frac{-2\pi }{\lambda }\sum_{k}d_{k}(x)(\beta_{k}(\lambda )\pm \Delta \beta_{k}(\lambda ))\right)\\ \cdot \;\text{exp}\left(\frac{-2\pi i}{\lambda }\sum_{k}d_{k}(x)(\delta_{k}(\lambda )\pm \Delta \delta_{k}(\lambda ))\right)\\ \cdot {\left[\text{exp}\left(\frac{2\pi i}{\lambda }\sum_{l}d_{l}\left(i\beta_{l}(\lambda )-\delta_{l}(\lambda )\right)\right)\right]}^{-1}. \end{array}$$ (A1)

With the last factor, we normalize the transmission function to the EO-generated absorption and phase shift of all laterally homogeneous layers *l* with thickness *d_l_* such as the substrate as well as the magnetic film and its seed and capping layers. Finally, we account for the finite probing wavelength of λ = 20.8 nm by a Gaussian convolution, *G*(*x*). The width of the Gaussian kernel, *σ*, is the only free parameter in the simulation and was chosen such that the convolved transmission function,

$${\tilde{T}}^{\pm }(x)=T^{\pm }(x)*G(x),$$ (A2)

matches the experimentally observed Fourier coefficients. We retrieve $\sigma_{\text{on}}=9.3\;\text{nm}$ by simultaneously fitting all diffraction orders in good agreement with the expected diffraction limit (λ/2). Table I lists the optical constants in the XUV spectral range used in our quantitative model.

**TABLE I.** Theoretical EO and MO constants β,δ and Δβ, Δδ of Al, Al_2_O_3_, and Co at wavelengths of λ=17.8 nm and λ=20.8 nm with dispersive *δ* and absorptive *β* components.^32^

	17.8 nm (Off resonant)			20.8 nm (On resonant)		
Material	Al	${\text{Al}}_{2}O_{3}$	Co	Al	${\text{Al}}_{2}O_{3}$	Co
*β*	0.0021	0.0357	0.1262	0.00 234	0.054	0.1455
*δ*	−0.0107	0.046	0.0889	0.0082	0.0749	−0.0037
Δβ						0.01 465
Δδ						0.008

## APPENDIX B: OFF RESONANT MEASUREMENT

Off-resonant SAXS measurements were conducted statically on the one hand in order to better understand the initial static nanometer-sized Al grating that serves as the starting point for time-dependent investigations and on the other hand to rule out the possibility that a laser-induced refractive index change in Al contributes to the measured signal at the Co M edge. Figure 8(a) shows a typical diffraction pattern of the unexcited grating sample taken at a wavelength of ≈17.8 nm (69.5 eV). Due to the higher photon energy, higher scattering vectors up to the fifth diffraction order can be recorded in contrast to the measurement at the Co resonance. The fact that no scattering along $q_{y}$ of magnetic origin can be detected shows that the observed diffraction can clearly be assigned to the metallic Al grating. The Fourier coefficients extracted from Fig. 8(a) are compared in Fig. 8(b) with the identical model as in the on-resonant case (see Fig. 4), but using a convolution kernel width scaled to the lower wavelength ($\sigma_{\text{off}}=\sigma_{\text{on}}\cdot 17.8\;\text{nm}/20.8\;\text{nm}\approx 8\;\text{nm}$). The Fourier coefficients measured off-resonantly can also be reproduced excellently up to the fifth diffraction order. The striking differences between the Fourier coefficients measured off- and on-resonant result from the strongly energy-dependent absorption and phase shift caused by Al.

The time-dependent measurements did not reveal a dependence of the scattered intensity on the optical excitation so that we assume throughout this work that the laser-driven dynamics only affect the electro- and magneto-optical constants of the resonantly probed Co layers.

## APPENDIX C: FINITE-ELEMENT SIMULATION

This section closely follows the discussion of the doctoral thesis of Weder.^26^

A 2D simulation of the electrical field distribution using the RF module of the commercial grade simulator COMSOL Multiphysics based on the finite-element method is conducted. To model the excitation, we start with the peak excitation fluence,

$$F=\frac{E_{p}}{2\pi \sigma_{a}\sigma_{b}},$$ (C1)

for a Gaussian beam profile, with *σ*_a_ and *σ*_b_ describing the 2D beam profile on the sample with $\sigma_{a,b}={\text{FWHM}}_{a,b}/2\sqrt{2\;\text{ln}\;(2)}$ and the pulse energy $E_{p}$. For solving the Maxwell equation, we need the electric field

$$E=\sqrt{\frac{F}{\sqrt{2\pi }c\varepsilon_{0}\tau }},$$ (C2)

with *c* being the speed of light, *ε*_0_ the vacuum permittivity, and $\tau =\text{FWHM}/2\sqrt{2\;\text{ln}\;(2)}$ the pulse length. The linear polarization of the monochromatic wave, centered at λ=400 nm, is perpendicular with respect to the alignment of the Al grating lines. The direction of incidence is normal. The absorbed power

$$P_{\text{abs}}=-0.5\omega {\left|E\right|}^{2}ℑ(\varepsilon_{r})$$ (C3)

is obtained from the incident electric field strength, *E*, from Eq. (C2), the angular frequency, *ω*, and the relative permittivity, $\varepsilon_{r}={\left(n-ik\right)}^{2}$.

After modeling the excitation, we now need to model the sample properties. In Table II, the real and imaginary parts *n* and *k* of the index of refraction at λ=400 nm used for the simulation are listed. For reasons of simplification, the multilayer structure is assumed to be a homogeneous CoPd alloy. This approach is justified because the wavelength is many times greater than the individual monolayers. To determine the weighted refractive index, we start with the molar volume $V_{m}$ of each element and relate it to the proportional layer thickness $d_{l}$ listed in Table III. For the weighted real part of the refractive index, we determine

$$n=\frac{1}{1+\frac{V_{m}^{\text{Co}}}{V_{m}^{\text{Pd}}}\frac{d_{l}^{\text{Pd}}}{d_{l}^{\text{Co}}}}\cdot n_{\text{Co}}+\frac{1}{1+\frac{V_{m}^{\text{Pd}}}{V_{m}^{\text{Co}}}\frac{d_{l}^{\text{Co}}}{d_{l}^{\text{Pd}}}}\cdot n_{\text{Pd}}.$$ (C4)

For the absorbing part, the calculation follows analogously.

**TABLE II.** Real- and imaginary parts *n* and *k* of the index of refraction at λ=400 nm for all compounds of the sample. Note that the Co/Pd multilayer is treated as a homogeneous film with effective *n* and *k*.

Layer	${\text{Si}}_{3}N_{4}$	Co	Pd	CoPd	Al	Al_2_O_3_
*n*	2^33^	1.578^34^	1.306^34^	1.472^34^	0.375^35^	1.76^36^
*k*	0^33^	2.953^34^	2.95^34^	2.952^34^	4.23^35^	0.02^36^

**TABLE III.** For the determination of the weighted real- and imaginary part of the refractive index, the molar volumes $V_{m}$ and the individually summed up layer thicknesses $d_{l}$ are required.

	Co	Pd
$V_{m}\left(m^{3}\;\text{mo}l^{-1}\right)$	$6.67\times 10^{-6}$	$8.56\times 10^{-6}$
$d_{l}$ (nm)	12	6

After modeling the excitation and sample, the boundary conditions need to be specified. The calculation is done on a single unit cell with the periodic boundary condition and perfectly matched layers (absorbing layer) above and below the structure along the *z*-axis. The Maxwell equation is solved on nodes of a triangle mesh. The edge length ranges between 0.1 nm and 5 nm. The radius of the Al corners were set to 2 nm.

Figure 9(a) displays a 2D map of the absorbed power $P_{\text{abs}}$ for a model grating with a period of 221 nm. In Fig. 9(b), we additionally show the absorbed power integrated along the XUV propagation axis, which we later use as a basis for the 1D diffraction model. We find the modulation of the excitation as expected. In the areas with the highest field enhancement, localized below the metal/oxide interface, the magnetic film absorbs approximately five times more laser power compared to the uncovered areas. As a result, the structured excitation induces a transient spatially modulated electronic and magnetic pattern in the multilayer sample. In particular, this leads to alternating fully magnetized and (partially) demagnetized areas.

## APPENDIX D: DOUBLE-EXPONENTIAL FIT

To extract the time constants from the transient demagnetization curve used for modeling the magneto-optical constants of the dichroic index of refraction, we applied the widely excepted model^37^

$$\begin{array}{c} \frac{\Delta M(t)}{M_{0}}=M_{0}+[A_{\text{de}}\left(1-\text{exp}\;\left(\frac{-t}{\tau_{\text{de}}}\right)\right)\\ +\;A_{\text{re}}\left(1-\text{exp}\;\left(\frac{-t}{\tau_{\text{re}}}\right)\right)+A_{0}F(\tau_{0},t)]\Theta (t), \end{array}$$ (D1)

with step function Θ(t), amplitudes A, and time constants *τ* for de- and remagnetization (de and re), respectively. While the first term describes the normalized unexcited magnetization state $M_{0}=M(t<0)$, the second and third terms account for the ultrafast magnetization quenching and slower recovery of the remagnetization process. The last term $A_{0}F(\tau_{0},t)$ usually considers a diffusive heat flow compensating for the excitation gradient between excited and unexcited sample volumes, with *A*_0_ being the value of $\Delta M(t)/M_{0}$ after the subsystems of electrons, phonons, and spins have equilibrated. As the thermalization time constant is several orders of magnitude smaller $\tau_{0}\gg \tau_{\text{re}},\tau_{\text{de}}$, we neglect the aspect of thermalization in our case and set $A_{0}F(\tau_{0},t)=0$.

To account for the temporal width of the pump and probe pulse duration of 80 fs and 70 fs (FWHM), respectively, limiting the temporal resolution, we temporally blur M(t) by a Gaussian convolution to obtain the measured transient magnetization

$$\tilde{M}(t)=(M*G)(t).$$ (D2)

## APPENDIX E: SECOND-ORDER DIFFRACTION EFFICIENCY AND ITS STATISTICAL ANALYSIS

In Fig. 10, we show the measured (a) and calculated (b) second-order diffraction intensity for all gratings A–I. Close inspection reveals that with increasing periodicity, the laser-driven changes, $I(t)/I_{0}$, increase, reach a maximum for gratings E and F, and then start to decrease again for grating G. Within our noise limit, grating H does not show any clear transient changes, while $I(t)/I_{0}$ of grating I clearly drops below 1 after optical excitation. In order to quantify the confidence interval in which we can determine whether the second-order diffraction intensity increases or decreases after optical excitation, we have performed a Student's T test of the data shown in Fig. 5. We define two time intervals: the first comprises N = 13 points before optical excitation between −1.5 ps and 0 ps and the second comprises N = 16 data points and covers the time delay points after optical excitation between 0.1 ps and 1.0 ps. We calculate the mean value of $I(t)/I_{0}$ within these two intervals, $\bar{X}[\Delta t]$, and the corresponding error, Σ[Δt], according to

$$\Sigma [\Delta t]=\frac{\mathrm{STD}[\Delta t]}{\sqrt{N-1}}\cdot T,$$ (E1)

where STD[Δt] is the standard deviation of $I(t)/I_{0}$ within each time interval and T is the Student's test value for a given confidence interval. In Fig. 11, we plot the average value $\bar{X}[\Delta t]$ with a 3σ (0.9973) confidence interval corresponding to *T *=* *3.64. The values for time delays before the optical excitation (red circles) fluctuate around 1, as $I(t<0)\overset{!}{=}I_{0}$; their confidence level determines how well we can determine the static diffraction intensity before optical excitation. The average values recorded for times after optical excitation (blue squares) increase ($\bar{X}[\Delta t]>1$) for grating A–G and decrease ($\bar{X}[\Delta t]<1$) for grating I. Since the error bars of the two time intervals do not overlap, we can claim this with a confidence interval exceeding 3σ.
